# Multiomics comparative analysis of feces AMRGs of Duroc pigs and Tibetan and the effect of fecal microbiota transplantation on AMRGs upon antibiotic exposure

**DOI:** 10.1128/spectrum.01983-24

**Published:** 2024-11-29

**Authors:** Tao Wang, Yuheng Luo, Xiangfeng Kong, Ling Fang, Liping Zhu, Bing Yu, Ping Zheng, Zhiqing Huang, Xiangbing Mao, Yu Jie, Junqiu Luo, Hui Yan, Jun He

**Affiliations:** 1 Animal Nutrition Institute, Sichuan Agricultural University, Chengdu, Sichuan, China; 2 Key Laboratory of Animal Disease-resistant Nutrition, Chengdu, Sichuan, China; 3 Institute of Subtropical Agriculture, Chinese Academy of Sciences, Changsha, Hunan, China; 4 Zhucheng Haotian Pharmaceutical Co., Ltd, Zhucheng, Shandong, China; Nanjing Agricultural University, Nanjing, China

**Keywords:** metagenomic, metatranscriptome, antimicrobial resistance genes (AMRGs), fecal microbiota transplantation (FMT), pig, feces

## Abstract

Fecal matter is recognized as both a reservoir and a transmission source for various antimicrobial resistance genes (AMRGs). However, the transcriptional activity of AMRGs in swine feces is not well understood. In addition, the effect of fecal microbiota transplantation (FMT) on the excretion of AMRGs has rarely been reported. Our study explored the diversity, abundance, transcriptional activity, and bacterial hosts of AMRGs in Tibetan and Duroc pig feces using metagenomic and metatranscriptomic sequencing technologies. We discovered a significantly higher genomic abundance of AMRGs in the feces of Duroc pigs compared to Tibetan pigs (*P <* 0.001), although the transcript levels did not show a significant difference. The results showed that the core composition of AMRGs in pig feces varied considerably, with the most transcriptionally active AMRGs being *oqxB*, *tetQ*, *Bla1*, *dfrA1*, and *amrB*. Furthermore, the *Firmicutes* phylum is the main host of AMRGs. By transplanting fecal flora from Tibetan and Duroc pigs into the intestines of Duroc Landrace Yorkshire (DLY) piglets after acute antibiotic exposure, we found that only Tibetan pig fecal flora significantly reduced AMRGs in the feces of DLY piglets (*P <* 0.05). The effectiveness of Tibetan pig fecal microorganisms in removing AMRGs from DLY pig feces was mainly influenced by microbial communities, especially the *Bacteroidota* phylum. These findings offer valuable insights for the prevention and control of AMRG pollution.

**IMPORTANCE:**

To the best of our knowledge, this study represents the first comprehensive analysis of antimicrobial resistance gene (AMRGs) expression in the fecal microbiota of Tibetan and Duroc pigs, employing an integrated metagenomic and metatranscriptomic approach. Our findings indicate a higher risk of AMRGs transmission in the feces of Duroc pigs compared to Tibetan pigs. Given the escalating antimicrobial resistance crisis, novel therapeutic interventions are imperative to mitigate gut colonization by pathogens and AMRGs. In this regard, we investigated the impact of fecal microbiota from Tibetan and Duroc pig sources on AMRGs excretion in Duroc Landrace Yorkshire (DLY) piglets’ feces following acute antibiotic exposure. Remarkably, only fecal microbiota sourced from Tibetan pigs exhibited a reduction in AMRGs excretion in DLY piglets’ feces. This underscores the significance of evaluating the presence of AMRGs within donor fecal microbiota for effective AMRGs decolonization strategies.

## INTRODUCTION

Antibiotic resistance in pathogenic bacteria is a major global public health threat (1), resulting in nearly 5 million deaths annually worldwide due to antibiotic-resistant bacteria (2). The widespread use of antibiotics in food animals is a key factor driving the rapid increase in antimicrobial resistance globally (3). Pigs, being the primary meat source for human consumption, account for over 40% of all meat consumed worldwide (4). China, as the world’s largest pig producer and consumer of veterinary antimicrobials, is a significant hotspot for antimicrobial resistance (5). A recent survey revealed that pigs accounted for 52.2% of total antimicrobial drug usage in China (6). The transmission of antimicrobial resistance genes (AMRGs) through the ecological cycle is a growing threat to public health. A detailed study, assessing pigs across various regions, farming practices, and breeds in China using macro-genomic methods, highlighted that factors such as farm location, antimicrobial use, housing conditions, and rearing practices critically influence AMRG variations (7). Another study investigating *Escherichia coli (E. coli)* in 1,871 pig and environment samples found widespread multidrug resistance (8). While existing research has focused on AMRG production in pigs and their environments, the transcriptional activity of these genes is vital for both livestock production and environental safety. However, the expression of AMRGs in swine feces has received limited attention, primarily due to the challenges in processing fecal RNA samples. Gene expression is a more accurate measure of functional activity than gene abundance (9). Thus, integrating metagenomic and metatranscriptome analyses, along with genetic background and host identification, is crucial for a better understanding of the risks posed by fecal microbial colonies carrying AMRGs and for advancing our knowledge of environmental resistance.

Acute exposure to broad-spectrum antibiotics is a significant pathway for the development of antibiotic-resistant bacteria and the accumulation of AMRGs in the animal gut and feces (10). Biodegradation and fecal microbiota transplantation (FMT) have been demonstrated as effective methods for the removal of AMRGs (11, 12). FMT is increasingly used for treating recurrent *Clostridium difficile* infections, effectively restoring colonization resistance by enhancing beneficial anaerobic bacteria and reducing potential pathogens (13, 14). FMT has demonstrated efficacy in facilitating the decolonization of multidrug-resistant microorganisms, exhibiting decolonization rates ranging from 37.5% to 87.5% (15). Limited studies have corroborated that FMT treatment reduces the quantity and diversity of AMRGs in the feces of patients with recurrent *C. difficile* infection within a 1-year timeframe (16). A recent study also emphasized the notable reduction in antibiotic resistance gene load among non-cirrhotic patients following FMT (17). However, due to the transmissible nature of FMT therapy, variations in the abundance of AMRGs carried by the donor microbiota during the transplantation process can result in FMT not always effectively reducing antimicrobial-resistant organisms (AROs) and AMRGs in the recipient’s gut (18). Consequently, assessing the AMRGs present in the donor fecal microbiota and investigating the dynamic changes in AMRG abundance in the recipient’s feces before and after transplantation are critical for understanding the kinetics of AMRG implantation through FMT.

This study first comprehensively assessed the diversity, distribution, and transcriptional activity of resistomes in the feces of Tibetan and Duroc pigs under different feeding regimes through metagenomic and metatranscriptomic analyses. Additionally, the bacterial species integrating AMRGs were identified. Furthermore, we performed FMT using the fecal microbiota from Tibetan and Duroc pigs as donor microbes on Duroc Landrace Yorkshire (DLY) piglets following acute broad-spectrum antibiotic exposure. The impact of the fecal microbiota from Tibetan and Duroc pigs on the microbial composition and AMRG excretion in DLY pigs post-acute antibiotic exposure was investigated using qPCR and 16S rRNA gene sequencing. The findings provide valuable insights for the prevention and control of AMRGs.

## MATERIALS AND METHODS

### Animal trial and sample collection

**Fig 1:**
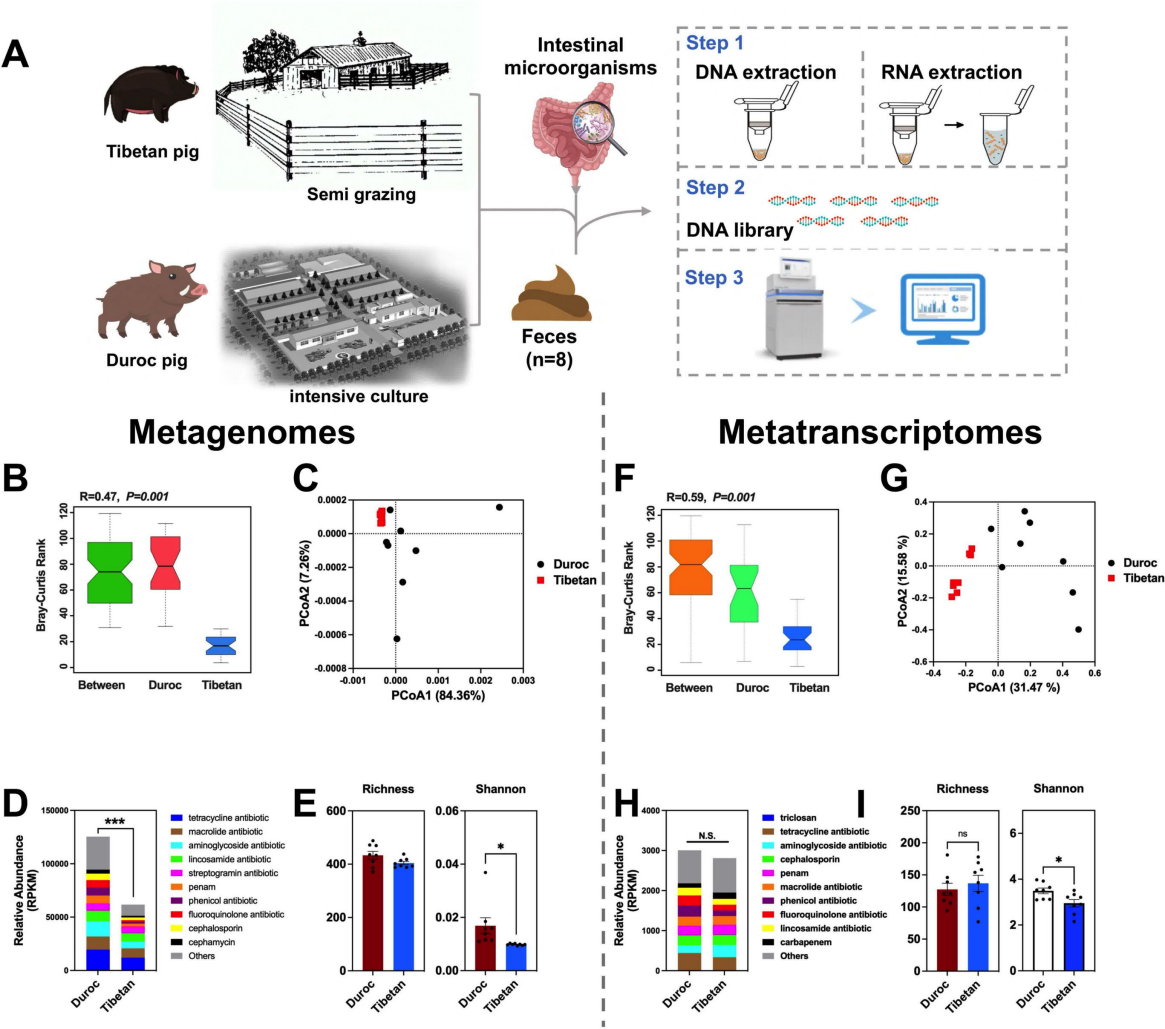
Integrated macrogenomic and macrotranscriptomic analysis of AMRG diversity in Tibetan and Duroc pig feces. (**A**) Flowchart of sampling analysis; (**B**) analysis of similarities (ANOSIM), (**C**) PCoA analysis, (**D**) relative abundance of total AMRGs, and (**E**) α-diversity of AMRGs in feces of Tibetan and Duroc pigs at the genome level. (**F**) ANOSIM, (**G**) PCoA analysis, (**H**) relative abundance of total AMRGs, and (**I**) α-diversity of AMRGs in feces of Tibetan and Duroc pigs at the transcript level. ***P  <  0.001, **P  <  0.01, *P  <  0.05.

The experiment began with the selection of physically mature Tibetan balsam pigs (boars, *n* = 8; body weight: 40–50 kg, semi-grazing farming, Ya'an) and Duroc pigs (boars, *n* = 8; body weight: 40–50 kg, intensive farming, Chengdu) and the collection of fresh, uncontaminated feces from the farms, which were frozen on dry ice (Fig. 1A). No antibiotics were used for 1 month prior to sampling. The feces were used for subsequent macro-genomic and macro-transcriptomic sequencing and faecal colony transplantation assays.

Thirty-three 21-day-old DLY piglets (ternary crossbred pigs: long white [large white], York and Duroc boars) were selected and randomly divided into four treatments according to the principle of homogenization of body weight and maternal origin, control: control (*n* = 8); antibiotic: antibiotic (*n* = 9); Duroc fecal flora transplantation: Duroc FMT (*n* = 7); and Tibetan pig fecal flora transplantation: Tibetan FMT (*n* = 9). At 1–14 days, control was gavage with saline seven times every other day, and the antibiotic, Duroc FMT, and Tibetan FMT were given 7 gavage of broad-spectrum antibiotics every other day. At 15–28 days, control and antibiotic were given 7 gavage of saline every other day, and Duroc FMT and Tibetan FMT were given 7 gavages of fecal suspensions of Duroc and Tibetan pigs, respectively, every other day. Fresh fecal samples were collected on days 14 and 28 for subsequent testing. The samples were stored in a refrigerator at −80°C. Composed of broad-spectrum antibiotics: ciprofloxacin 1 g (Northeast Pharmaceutical Group Co., Ltd) and oral metronidazole 5 g (Northeast Pharmaceutical Group Co., Ltd) (19, 20).

Preparation of fecal suspension: Feces from donor pigs of the same breed were mixed together, and 1,000 g of each was weighed under anaerobic (beakers were sealed during mixing), aseptic conditions, and added to sterile saline at a ratio of 1:3 (mass to volume ratio), and mixed by shaking. The fecal suspension was filtered through a filter (50 mesh, then 100 mesh) and stored at −80°C with 10% sterile glycerol.

Saline: Saline was added to L-cysteine hydrochloride solution (0.25 g/L) as a reducing agent and deoxygenated with nitrogen and autoclaved.

### Experimental procedures of metagenomic sequencing

Total DNA was extracted from the samples using the QIAamp Fast DNA Stool Mini Kit (QIAGEN, Germany) according to the manufacturer’s instructions. The purity and integrity of the DNA were analyzed using a 1% agarose gel electrophoresis (AGE) assay, and DNA was quantified using a Qubit dsDNA Assay Kit in Qubit 2.0 Flurometer (Life Technologies, CA, USA) for DNA quantification. One microgram of genomic DNA from the samples was used for library construction using NEBNext Ultra DNA Library Prep Kit for Illumina (NEB, USA). After the library was constructed, Qubit 2.0 was used for preliminary quantification, and the library was diluted to 2 ng/µl. Then the insert size of the library was detected using an Agilent 2100, and after the insert size met the expectation, the effective concentration of the library was accurately quantified using the quantitative PCR (qPCR) method (the effective concentration of the library was more than 3 nM) to ensure the quality of the library quality. The libraries were sequenced on the Illumina HiSeq platform. Sequence quality control was performed by a pre-programmed procedure, and if the samples were contaminated with host sequences, the samples were aligned with the host sequences to filter out the reads that might be of host origin (21–23) (by default, Bowtie2 software was used, with the following parameters: --end-to-end, --sensitive, -I 200, and -X 400). After pre-processing, clean data were obtained, and assembly analysis was performed using MEGAHIT assembly software (assembly parameters: --presets meta-large). Comparison with functional databases using genes, DIAMOND software (24) compared Unigenes with Bacteria, Fungi, Archaea, and Viruses sequences extracted from non-redundant Database (NR) of the National Center for Biotechnology Information (NCBI) (Version: 2018.01) database (blastp, *e* ≤ 1^e−5^) (21). A comprehensive antibiotic resistance database (CARD v3.2.5, https://card.mcmaster.ca/) was downloaded and organized, and the AMRG database was constructed using DIAMOND software (v2.0.15.153) (24). The open reading frame (ORF) files were compared with the AMRG database using DIAMOND software with the following parameters: *e =* 1^e−7^, max-target-seqs 70, id 80. AMRG-like ORFs were identified. Clean macrogenomes of all samples were analyzed using Metaspades (v3.14.0) with default parameters The data were finely assembled, and the macrogenome-assembled genome (MAG) was assembled using overlapping clusters (25). The MAG was generated from Metabat2, Maxbins, and CONCOCT in the MetaWRAP pipeline (26). Species classification of MAGs was performed using GTDB-TK v2.1.1 and the GTDB database (R207_v2) (27, 28). The raw data were uploaded to National Genomics Data Center (Accession number: CRA014735).

### Experimental procedures for metatranscriptome sequencing

RNA library for metatranscriptome-seq was prepared as rRNA depletion and stranded method. Briefly, the ribosomal RNA was depleted from total RNA using the rRNA Removal Kit following manufacturer’s instruction. RNA was then fragmented into 250- to 300-bp fragments and reverse-transcribed into cDNA subsequently. After adenylation of 3′ ends of DNA fragments, sequencing adaptors were ligated to the cDNA. In order to select cDNA fragments of preferentially 250–300 bp in length, the library fragments were purified with AMPure XP system. Amplification of cDNA was performed using PCR.

After library construction, metatranscriptome sequencing was performed using the Illumina platform to obtain 5/10 G of raw data. The raw data in FASTQ format (raw reads) is first processed by an internal perl script. Use DIAMOND software to compare unigenes with the sequences of bacteria, fungi, archaea, and viruses extracted from NCBI’s NR ([Version: 2016–11-05] database [blastp], *e* ≤ 1^e−5^) (24). According to the LCA annotation results and the gene abundance table, the abundance information and the table of gene numbers of each sample at each taxonomic level (genus, phyla, family, genus, and species) were obtained. To obtain functional information of AMRGs carried by microorganisms, gene function annotations were retrieved from the CARD database. Using DIAMOND software, unigenes were mapped to each functional database (blastp, *e* ≤ 1^e−5^) (24). The raw data were uploaded to NCBI SRA database (sequence number: PRJNA1077706).

### qPCR

DNA was extracted from fecal samples according to the instructions of the Fecal Genomic DNA Extraction Kit (D2700, Solarbio, Beijing, China). Concentration and integrity of DNA samples were checked by a Qubit Ultra Microspectrophotometer (NanoDrop 2000, Thermo Scientific, Waltham, MA, USA) and gel imaging (GelDocXR, Bio-Rad, Hercules, CA, USA) to detect the concentration, purity, and integrity of the DNA samples. Fecal DNA was used as the template for qPCR quantitative analysis of specific AMRGs (CFX96 Real-Time Fluorescence Quantification Instrument, ABI7900, USA). Ten microliters of reaction system was 5 µL of SYBR Premix Ex TaqTM II, 0.5 µL each of the upstream and downstream primers, 2 µL of DNA template, and 2 µL of ddH_2_O. Amplification procedure: pre-denaturation at 98°C for 1 min; denaturation at 98°C for 10 s, annealing at 60°C for 30 s, extension at 72°C for 30 s for a total of 30 cycles, and extension at 72°C for 5 min. The abundance of 20 genes in the fecal samples was examined using qPCR (QS6FX, ABI, Waltham, MA, USA) (Table S1). A total of 20 genes included six pairs of tetracyclines (*tetA*, *tetC*, *tetM*, *tetQ*, *tetO*, and *tetX*), one aminoglycoside (*strB*), two chloramphenicol analogs (*cmlA* and *fexA*), two sulfonamides (*sul1* and *sul2*), one β-lactams (*blaTEM*), two macrolides (*ermB* and *ermC*), four (flor)/(chloror)/(am) phenolics (FCAs) (*qnrS*, *oqxA*, *cfr*, and *oqxB*), and the drug-resistant *intI1* and 16S rRNA genes. Absolute abundance of AMRGs was calculated according to Yang et al. (29). Normalized abundance of AMRGs was calculated according to Yang et al. (2021) (29).

### Analysis of the bacterial community

The total genomic DNA of the microbial community was extracted according to the instructions of the E.Z.N.A. Soil DNA kit (Omega Bio-tek, Norcross, GA, USA), the quality of the extracted genomic DNA was checked by AGE with 1% agarose, and the concentration and purity of the DNA were determined by NanoDrop 2000 (Thermo Scientific, USA). The quality of the extracted genomic DNA was measured by AGE (1%), and the DNA concentration and purity were determined by NanoDrop 2000 (Thermo Scientific). PCR amplification of the V3-V4 variable region of the 16S rRNA gene was performed using the above extracted DNA as a template using upstream primer 338F (5′-ACTCCTACGGGGAGGCAGCAG-3′) and downstream primer 806R (5′-GGACTACHVGGGTWTCTAAT-3′) (30), both of which carry barcode sequences. Library construction of purified PCR products was performed using the NEXTFLEX Rapid DNA-Seq Kit. Sequencing was performed using the Illumina platform (Shanghai Meiji Biomedical Technology Co., Ltd.). The double-ended raw sequencing sequences were quality controlled using fastp (31) (https://github.com/OpenGene/fastp, version 0.19.6) software, and spliced using FLASH (32) (http://www.cbcb.umd.edu/software/flash, the version 1.2.11) software for splicing. Operational taxonomic unit (OTU) clustering and chimera removal were performed on the quality control (QC) spliced sequences based on 97% similarity using UPARSE v7.1 (33) software (http://drive5.com/uparse/). OTU species taxonomy was annotated using the ribosomal database project (RDP) classifier (34) (http://rdp.cme.msu.edu/, version 2.11) compared to the Silva 16S rRNA gene database (v138), with a confidence threshold of 70%, and the community composition of each sample was counted at different species classification levels. The raw data were uploaded to NCBI SRA database (sequence number: PRJNA1071271).

### Statistics

Data pre-processing was performed using Excel 2019 (Microsoft, Washington, DC, USA), and data statistics were performed using SPSS 22.0 (IBM Corp, New York, NY, USA). Results were graphically displayed using GraphPad Prism 10. Reads Per Kilobase per Million mapped reads (RPKM) values were used for heat map data, *z*-score was used for data normalization, and mean clustering was used for cluster analysis. Correlation analysis was performed using the person method. The “protest” function in the vegan software package was used to analyze the correlation of Procrustes between the bacterial and resistance groups. Principal coordinate analysis (PCoA) and non-metric multidimensional scaling (NMDS) analyses were performed using the Bray–Curtis distance algorithm using the AMRG and normalized abundance values of the bacterial communities using the vegan package in R software (R version 4.0.2). Phylogenetic trees were plotted using ChiPlot (https://www.chiplot.online/) (35). Results were expressed as means ± standard error, *P <* 0.05 indicated a significant difference, and 0.05 *< P <* .1 is considered a trend of difference.

## RESULTS

### Multiomics analysis of the diversity of fecal AMRGs in Tibetan and Duroc pigs

Macrogenomic analysis delineated the genomic diversity of AMRGs in the feces of Tibetan and Duroc pigs under different breeding practices. Similarity analysis revealed marked differences in AMRG profiles between Duroc and Tibetan pig feces (*R* = 0.47, *P =* 0.001) (Fig. 1B). Principal component analysis (PCA) further underscored these substantial genomic differences in the AMRG structure of both pig breeds (Fig. 1C). Notably, the genomic abundance of AMRGs was significantly higher in Duroc pig feces than in Tibetan pig feces (*P <* 0.0001) (Fig. 1D). Alpha diversity analysis indicated a greater genomic-level Shannon index for AMRGs in Duroc pig feces compared to Tibetan pig feces (*P <* 0.05) (Fig. 1E).

Through metatranscriptome sequencing, the diversity of AMRG transcriptomes in the feces of both pig breeds under varied breeding practices was investigated. Similarity analysis showed significant transcriptomic differences in AMRGs between Duroc and Tibetan pig feces (*R* = 0.59, *P =* 0.01) (Fig. 1F and G). PCA results confirmed these notable differences in the AMRG transcriptome structure (Fig. 1G). The overall relative abundance of AMRG transcriptomes in Duroc and Tibetan pig feces did not differ significantly (Fig. 1H). However, alpha diversity analysis revealed a higher Shannon index for AMRG transcriptomes in Duroc pig feces than in Tibetan pig feces (*P <* 0.05) (Fig. 1I).

### Differences in the composition and transcriptional activity of AMRGs in the feces of Tibetan and Duroc pigs

**Fig 2:**
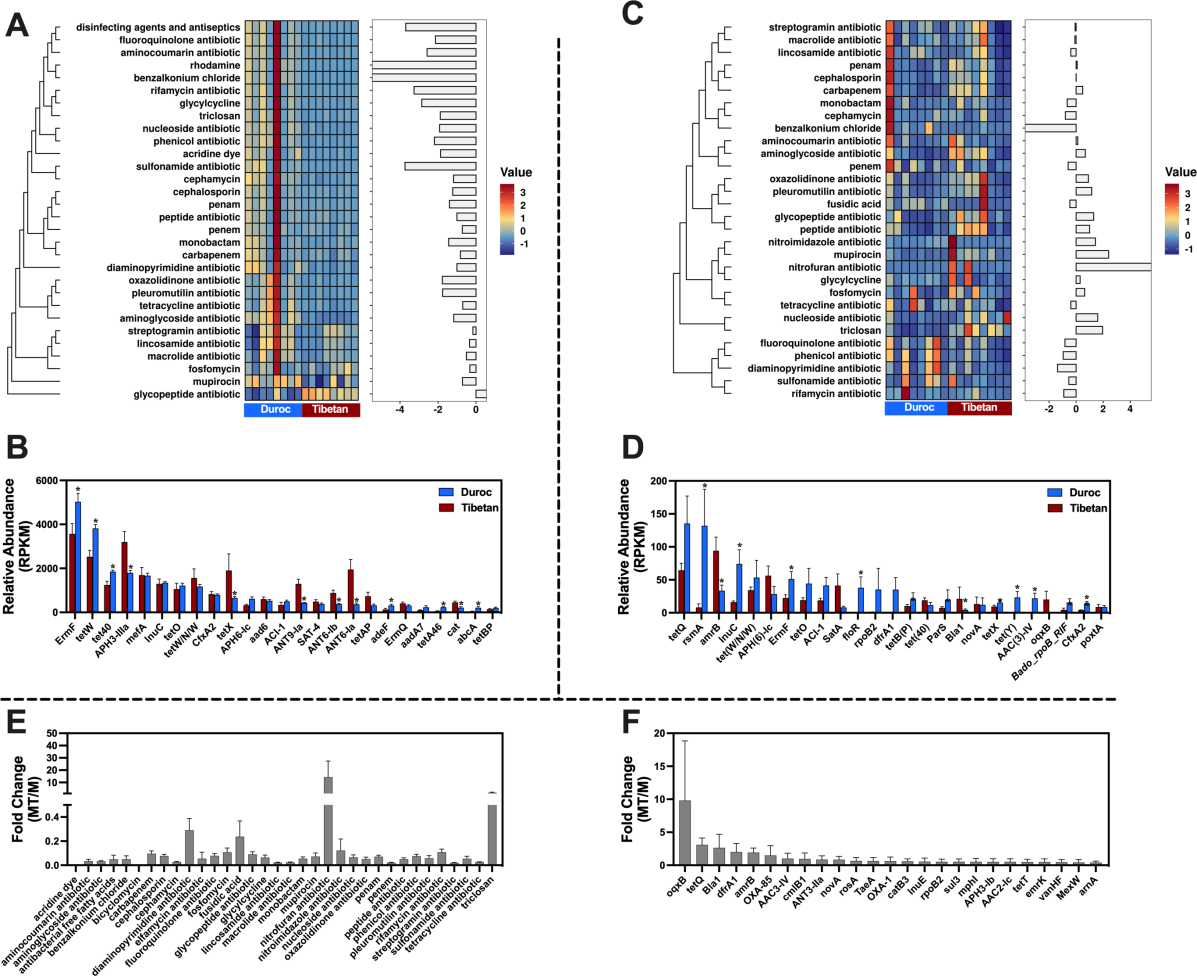
Integrated macrogenomic (**M**) and macrotranscriptomic (MT) analysis of AMRGs composition and transcriptional activity in Tibetan and Duroc pig feces. Heatmap of relative abundance of AMRGs per antimicrobial (M: A; MT: C). The left bar chart shows the abundance ratio of AROs in Tibetan pigs/Duroc pigs. Core AMRGs in feces of Tibetan and Duroc pigs (M: B; MT: D). Transcriptional activity of AMRGs (**E**) and Top20 AMRGs (**F**) for each antimicrobial. *P  <  0.05.

Metagenomic and metatranscriptome analyses identified 737 and 646 AMRGs in the feces of Tibetan and Duroc pigs, respectively. These AMRGs exhibited resistance to 30 commonly used antibiotics in humans and animals (Fig. 2A and C). Intriguingly, both analyses showed that tetracycline and aminoglycoside resistance genes were most abundant in the feces of both pig breeds (Fig. 2A and C). However, there were significant differences in the core composition of AMRGs as identified by the two sequencing methods. Metagenomic sequencing revealed that *ErmF*, *tetW*, *tet40*, *APH3-IIIa*, and *mefA* were predominant in the feces of both pig breeds. Notably, *ErmF, tetW*, and *tet40* had a significantly higher relative abundance in Duroc pigs compared to Tibetan pigs (*P <* 0.05), whereas APH3-IIIa was significantly lower in Duroc pigs (*P <* 0.05) (Fig. 2B). Transcriptomic sequencing indicated that the primary AMRGs were *tetQ*, *rsmA*, *amrB*, *lnuC*, and *tet (W/N/W)*, with *rsmA* and *lnuC* being significantly more abundant in Duroc pigs (*P <* 0.05) and *amrB* significantly less abundant (*P <* 0.05) (Fig. 2D).

We also evaluated the transcriptional activity of AMRGs in the feces of Tibetan and Duroc pigs based on fold changes in transcript and gene abundance. The highest transcriptional activity of total AMRGs was found for nitrofuran antibiotic resistance, triclosan, diaminopyrimidine antibiotic, and fusidic acid antibiotics, and the highest transcriptional activity was found for AMRGs such as *OqxB*, *tetQ*, *Bla1*, *dfrA1*, and *amrB* (Fig. 2E and F).

### Characterization of the host microbial composition of AMRGs

**Fig 3:**
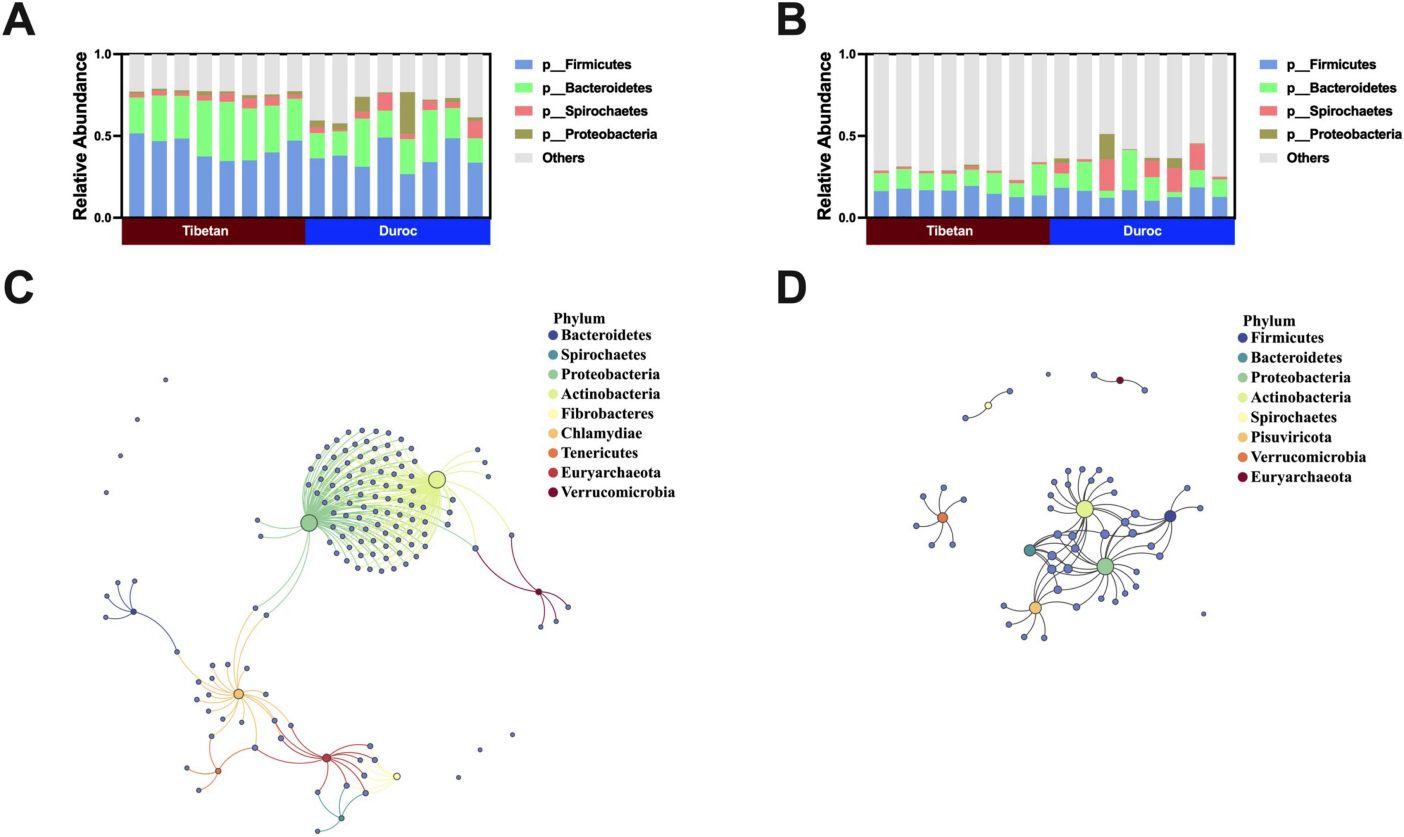
Microbial composition of feces from Tibetan and Duroc pigs. Phylum-level microbial composition of feces from Tibetan and Duroc pigs based on (**A**) macrogenomic and (**B**) macrotranscriptomic analyses. Co-occurrence patterns of AMRGs and bacterial communities in feces of Tibetan and Duroc pigs based on (**C**) macrogenomic and (**D**) macrotranscriptomic analyses.

Results from both metagenomic and metatranscriptome analyses consistently highlighted *Firmicutes*, *Bacteroidetes*, *Spirochaetes*, and *Proteobacteria* as the predominant phyla in the feces of Tibetan and Duroc pigs (Fig. 3A and B). Procrustes analysis, based on Bray–Curtis distance, demonstrated a strong alignment and significant correlation between the entire profile of AMRGs and the microbial community composition (Metagenomic: *M*_2_ = 0.8364, *P =* 0.001; metatranscriptome: *M*_2_ = 1.1564, *P =* 0.001) (Fig. S1). These findings suggest that the variations in AMRGs carried in feces align with the trends in microbial community changes, implying that diverse AMRGs may originate from distinct microbial sources. Further analysis supported this hypothesis. We conducted Pearson correlation analysis between AMRGs and microbial phyla, selecting highly significant and strongly correlated relationships (0.7 < |*r*| < 1, *P <* 0.05) to demonstrate co-occurrence networks. The results, confirmed at both genomic and transcriptomic levels, underscored the significant enrichment of AMRGs in potential hosts within the microbial community (Fig. 3C and D).

**Fig 4:**
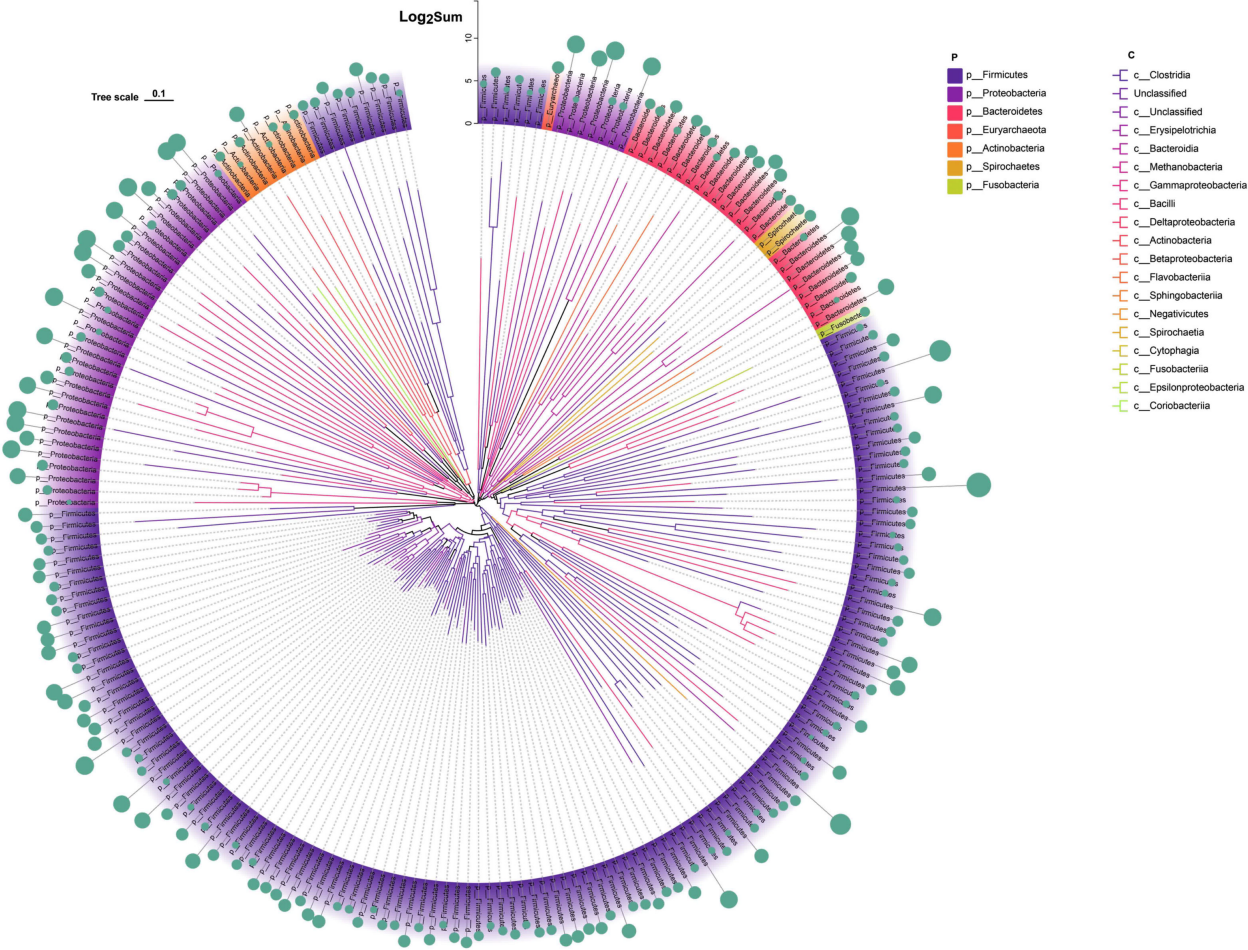
Number and abundance of AMRGs carried by MAGs in the feces of Tibetan and Duroc pigs.

To further confirm the presence of AMRG-carrying hosts in pig feces, metagenomic assembly and binning were performed on environmental samples. Following strict criteria for completeness and contamination, 217 MAGs were retained. These MAGs were identified using GTDB-TK for species classification and subjected to phylogenetic tree analysis. They spanned seven bacterial phyla, with the highest abundance in *Firmicutes* (63.5%), followed by *Proteobacteria* (29.4%), and *Bacteroidetes* (6.5%) (Fig. 4; Fig. S2). Out of the 217 MAGs, 165 were successfully annotated at the class level, including *Clostridia* (*n* = 84), *Gammaproteobacteria* (*n* = 24), *Bacteroidia* (*n* = 18), and *Bacilli* (*n* = 17) (Fig. 4).

### Effect of fecal flora transplantation on fecal AMRGs after acute antibiotic exposure

**Fig 5:**
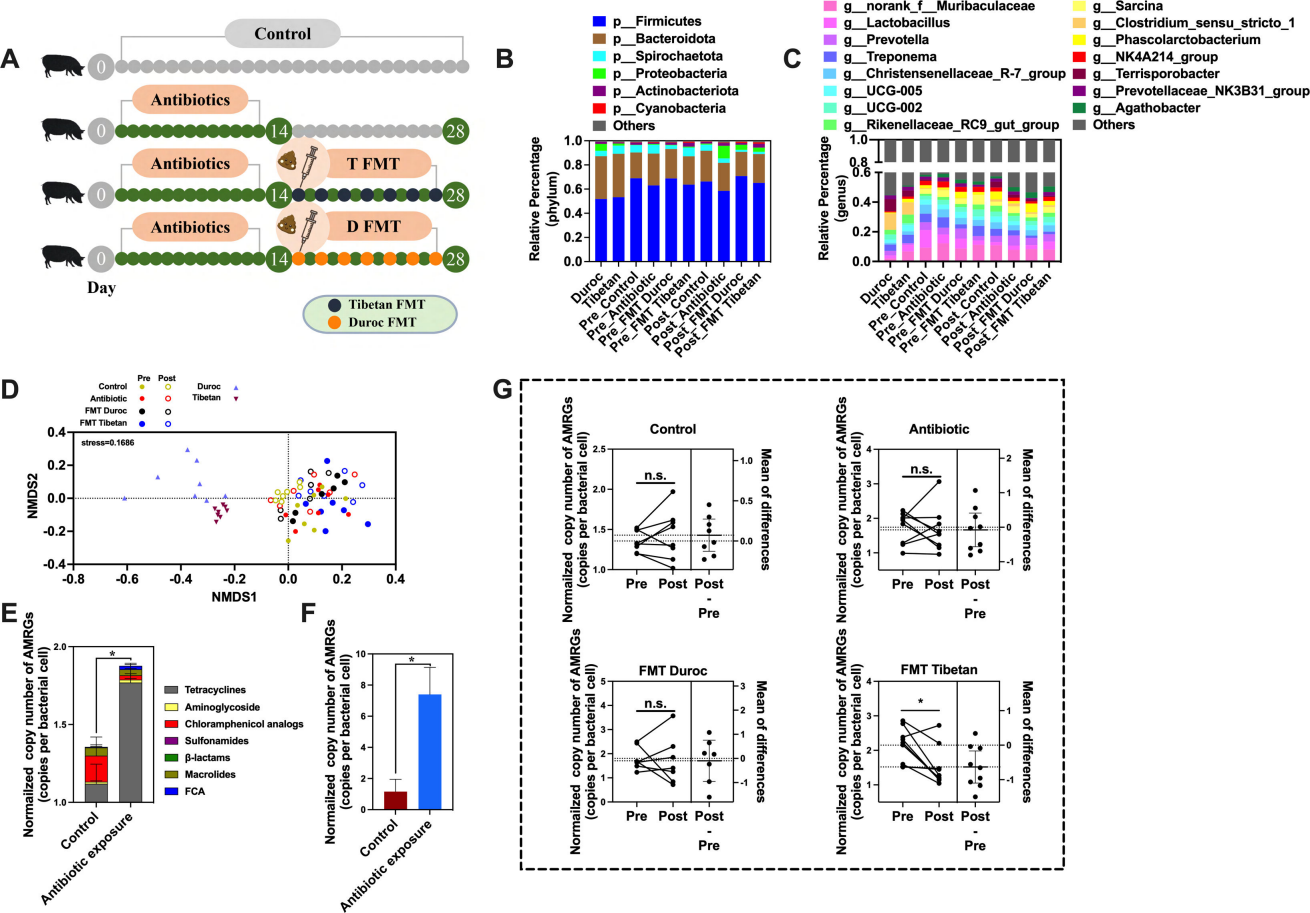
Effect of fecal flora transplantation on AMRGs in feces of DLY pigs after acute exposure to antibiotics. (**A**) Experimental pattern diagram. (**B**) Microbial composition at the phylum-level, (**C**) microbial composition at the genus level, and (**D**) PCoA analysis of microbes. Effect of acute exposure to antibiotics on (**E**) AMRGs and (**F**) int1 abundance in feces. (**G**) Dynamics of AMRGs abundance before and after fecal flora transplantation. *P  <  0.05.

To investigate the dynamic changes in AMRGs in the feces of DLY piglets after FMT from Tibetan and Duroc pigs following acute broad-spectrum antibiotic exposure, we initially subjected the antibiotic group, FMT Tibetan group, and FMT Duroc group to 14 days of broad-spectrum antibiotic gavage, administered every other day. Subsequently, the FMT Tibetan group and FMT Duroc group underwent 14 days of FMT, also performed every other day. Fecal samples were collected on the 14th and 28th days (Fig. 5A). To comprehend the changes in the fecal microbiota of DLY piglets after antibiotic exposure and FMT, we conducted 16S sequencing on fecal samples. Notably, the relative proportions of phylum-level microorganisms changed significantly before and after FMT, especially in the genera *Spirochaetota* and *Proteobacteria*, and among the genus-level microorganisms, significant changes were also observed in *Lactobacillus*, *Prevotella*, and *Treponema* (Fig. 5B and C). Characterization of fecal microbial community structure was performed using NMDS and permutational multivariate analysis of variance. The results revealed a distinct shift in the microbial community structure in DLY piglet fecal samples after FMT from Tibetan and Duroc pigs (Fig. 5D).

**Fig 6:**
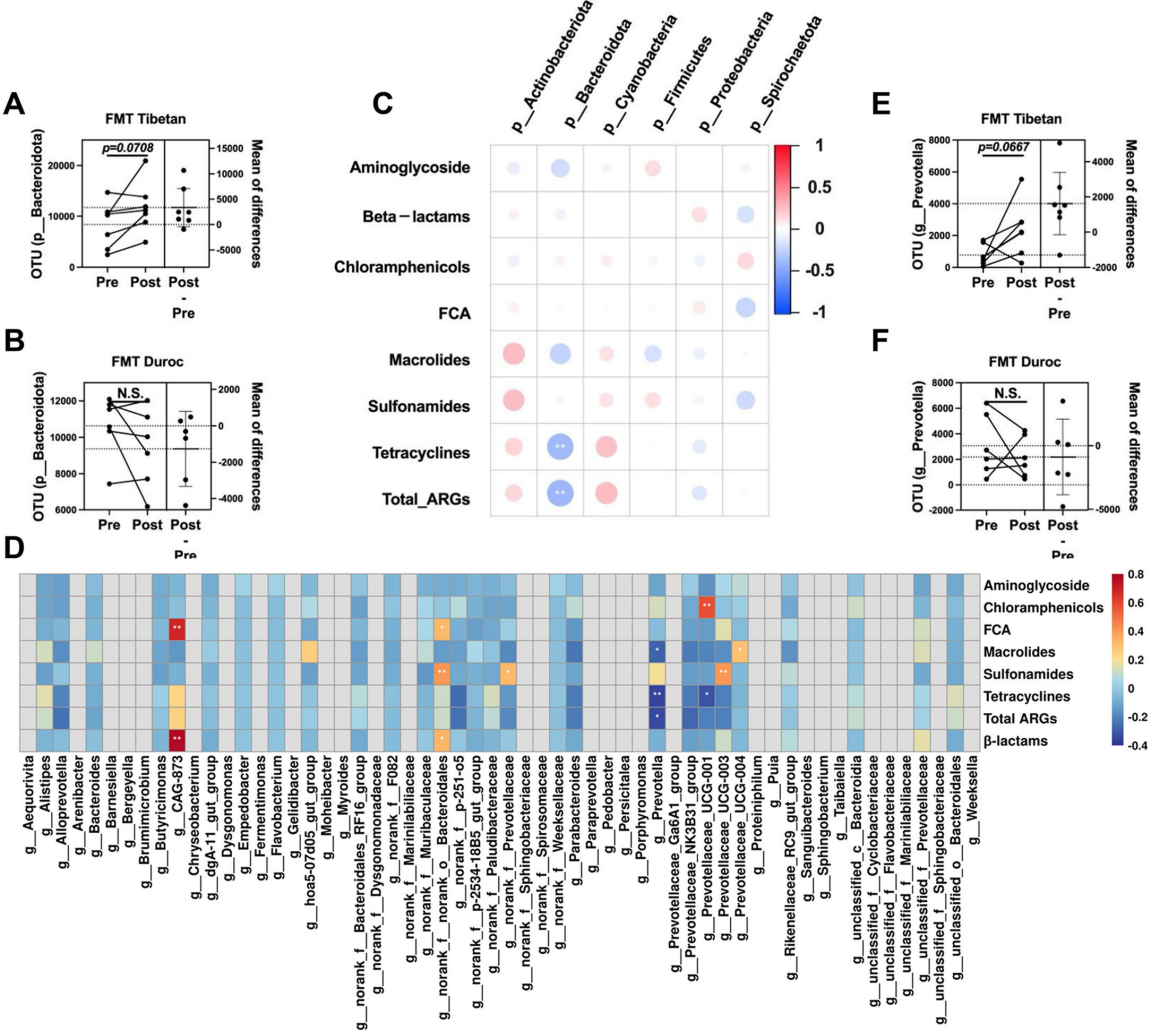
Drivers of fecal flora transplantation affecting excretion of AMRGs. (**A–B**) Dynamics of *p__Bacteroidetes* OUT levels before and after fecal flora transplantation. (**C**) Correlation analysis of gate level microorganisms with the abundance of AMRGs from different antimicrobial classes. (**D**) Correlation analysis of genus-level microorganisms of the phylum *Bacteroidetes* with the abundance of AMRGs of different antimicrobial classes. (**E–F**) Dynamics of *Prevotella* OUT levels before and after fecal flora transplantation. **P  <  0.01, *P  <  0.05.

To further explore the dynamic changes in AMRGs during the experimental process, we performed absolute quantification of common AMRGs in the collected samples. Initially, we compared the abundance of AMRGs and *int1* in the feces of DLY pigs between the control group and the antibiotic exposure group. We observed a significant increase in the abundance of AMRGs and *int1* in the feces of DLY pigs after antibiotic exposure (*P < 0.05*) (Fig. 5E and F). Furthermore, through paired analysis comparing the dynamic changes in AMRGs in feces at 14 days and 28 days among different groups, we found that only the Tibetan pig FMT group exhibited a significant decrease (*P <* 0.05) (Fig. 5G). By examining the changes in phylum-level microbial OUTs in fecal samples from different groups at 14 days and 28 days, we noted an increasing trend in *Bacteroidetes* in the Tibetan pig FMT group (0.05 *< P <* .1) (Fig. 6A and B; Fig. S3A through F). Subsequently, we conducted a Pearson correlation analysis between AMRGs and phylum-level microbes, confirming a significant negative correlation between *Bacteroidetes* and the abundance of total AMRGs as well as Tet genes (Fig. 6C). To further identify which genus-level microorganisms within *Bacteroidetes* might be crucial factors influencing AMRGs, we performed a Pearson correlation analysis between AMRGs and genus-level microorganisms belonging to *Bacteroidetes*. The results revealed a significant negative correlation between *Prevotella* and the abundance of total AMRGs, tetracyclines genes, and macrollines genes (Fig. 6D). Therefore, *Prevotella* may be a key driver in the reduction of AMRGs in the feces of DLY pigs after acute antibiotic exposure following Tibetan pig FMT. Paired analysis also confirmed an increasing trend in *Prevotella* after Tibetan pig FMT (*0.05 < P <* .1) (Fig. 6E and F; Fig. S3G and H).

## DISCUSSION

This study represents the first comprehensive comparison of AMRGs present in the feces of Tibetan and Duroc pigs, employing both metagenomic and metatranscriptomic analyses. While previous research has provided a genomic-level overview of AMRGs in various pig farms under different breeding conditions and scales, investigations into the transcriptional differences of AMRGs among different pig breeds, as well as the use of multiomics approaches to study the transcriptional activity of AMRGs in pig feces, remain scarce (7, 36). At the genomic level, our findings indicated that both the abundance and diversity of AMRGs in Tibetan pig feces were significantly lower than in Duroc pigs, aligning with earlier studies (7). However, at the transcript level, the diversity of AMRGs in Tibetan pig feces was significantly lower than that in Duroc pigs, with no significant difference in the relative abundance of AMRGs. This suggests that AMRGs at the genomic and transcript levels do not exhibit complete consistency. This could be attributed to the relatively stable microbial species carrying AMRGs encountered by different pig breeds in a fixed environment, coupled with the presence of a substantial number of deceased microbial bodies in pig feces.

Generally, the predominant classes of AMRGs found in swine feces are those conferring resistance to tetracyclines and aminoglycosides (7). Tetracycline resistance, in particular, is commonly observed in swine farms globally, including in China, the United States, and Europe (37–39). Interestingly, our study revealed that while significant differences exist in the core AMRGs in swine feces at both genomic and transcript levels, tetracycline resistance genes consistently showed core levels in both analyses. This pattern can be partially attributed to the long-term use of tetracycline in pig production (40). Notably, although *oqxB* and *Bla1* were not initially dominant at the genomic and transcriptomic levels, they exhibited the highest transcriptional activity when evaluating different AMRG classes by the ratio of transcript abundance to genome abundance. And these high transcriptional activity AMGRs have been proven to be important antibiotic resistant factors in pathogenic bacteria. For example, OqxB has undergone horizontal gene transfer and is now visible in other Gram negative bacterial pathogens, including *Escherichia coli*, and *Salmonella*, further spreading multidrug resistance (41). This finding is significant for assessing the actual activity of AMRGs in real-life environmental scenarios, highlighting potential gaps between gene abundance and active expression.

The dominant phylum-level microorganisms in swine feces, specifically *Firmicutes*, *Bacteroidetes*, *Spirochaetes*, and P*roteobacteria*, are consistent with our findings (42). Procrustes analysis and Pearson correlation analysis were employed to map the co-occurrence network, confirming that these predominant microorganisms in feces are potential hosts of AMRGs. However, further species annotation of contigs containing AMRG sequences is required, and the correlation of microorganisms with AMRGs needs to be determined. In line with prior studies, we identified Phylum *Firmicutes* and Phylum *Ascomycota* as the primary phylum-level microorganisms carrying AMRGs (43, 44). These hosts, predominantly carrying AMRGs, are susceptible to horizontal gene transfer among different bacteria associated with pig feeding environments and feeders. Evidence indicates that the gut microbiome and resistance profile can undergo remodeling in long-term farm workers and pet owners (44–46). Of greater concern to us is the fact that the core AMRGs host identified in this study, *c__Clostridia*, contains pathogenic bacteria, including *Clostridium tetani*, *Clostridium perfringens*, *Clostridium botulinum*, and *C. difficile*, which may pose a more complex risk of antibiotic resistance and pathogenicity (47). Admittedly, the initial phase of our study faces certain limitations, including a smaller sample size than desired. This was due in part to the challenges of collecting fecal samples from semi-grazing Tibetan pigs and the high costs associated with multiomics studies. Despite these constraints, the results we have obtained offer significant reference value and contribute to the understanding of AMRG dynamics in swine feces.

Our results show that acute antibiotic exposure significantly increases the normalized abundance of AMRGs and the integrase gene *intI1* in pig feces. This further confirms that antibiotics promote the enrichment and spread of AMRGs in animal feces (48). The DLY pig is a typical lean-type breed from Europe, widely used in China due to its fast growth rate, high meat quality, and superior feed conversion efficiency (49). Previous studies have shown that commercial pigs in China contribute to the spread of AMRGs to soil, air, and humans through pig farming environments, manure fertilization, and meat products (50–52). Therefore, we selected DLY piglets as the recipient model for FMT to explore potential strategies for reducing the risk of ARGs carriage and transmission in commercial pigs. However, in our study comparing FMT from Tibetan and Duroc pigs in antibiotic-exposed DLY piglets, we noted a significant reduction in AMRG abundance in the recipient feces only with Tibetan pig fecal flora. It has been demonstrated that transplantation of healthy and lower AMRGs-carrying donor fecal flora reduces the relative abundance of AMRGs carried by recipient fecal flora (12, 53, 54). However, a study of five patients with Crohn’s disease who underwent FMT found that three of them had increased total abundance of AMRGs (55). Similarly, DeFilipp et al. (56) reported that two patients who received FMT from the same donor became infected with Escherichia coli producing extended-spectrum beta-lactamases. These cases suggest that FMT may facilitate the transfer of AMRGs between donors and recipients, potentially promoting the horizontal transmission of pathogens. This also indicates that the higher abundance of AMRGs in the fecal microbiota of Duroc pigs could be the reason why Duroc-derived microbiota failed to reduce the ARG abundance in the feces of DLY piglets. Therefore, during FMT, it is critical to carefully screen donor fecal microbiota and their associated AMRGs to ensure the removal of AMRGs and prevent the colonization of AROs.

By examining changes in microbial dynamics at the phylum level before and after FMT, we observed that only fecal flora from Tibetan pigs significantly altered the OTUs levels of the *Bacteroidetes* phylum. This indicates that Bacteroidetes may play a key role in decreasing AMRG abundance in recipient feces through Tibetan FMT. Bacteroidetes, known for their beneficial properties, have extensive capabilities for digesting dietary fiber polysaccharides and host glycans (57). They also produce short-chain fatty acids, maintaining gut flora balance and exhibiting anti-inflammatory effects (57). Previous lab studies have also confirmed the potential of short-chain fatty acids to remove AMRGs carried by intestinal microbiota (43). It’s noteworthy that *Prevotella*, belonging to the *Bacteroidetes* phylum, showed an elevation in the feces of DLY pigs after Tibetan pig FMT. A study on African children following a low-fat, high-fiber diet demonstrated an enrichment of intestinal *Prevotella*.^58^ This further confirms that Tibetan pig FMT may reduce AMRGs levels in recipient feces by enhancing the intestinal flora’s ability in DLY piglets to digest fiber and promoting the colonization of beneficial bacteria.

### Conclusion

The study indicates that there is not complete concordance in the abundance of AMRGs between the genomic and transcriptomic levels in feces of Tibetan and Duroc pigs. However, overall, Duroc pigs exhibit a higher risk of AMRGs transmission compared to Tibetan pigs. It is noteworthy that the *Firmicutes* phylum predominates among microorganisms carrying AMRGs in feces. Additionally, through the transplantation of fecal microbiota from Tibetan and Duroc pigs into the intestinal tract of DLY piglets after acute antibiotic exposure, it was observed that the fecal microbiota of Tibetan pigs significantly reduces the content of AMRGs in DLY pig feces, with the removal effect primarily driven by the microbial community, especially the influence of the *Bacteroidota* phylum. These findings provide crucial insights for further combating AMRGs contamination, emphasizing the pivotal role of microbial communities in the removal of AMRGs from feces. Future research should delve deeper into this field to develop more effective prevention and control strategies, safeguarding both the environment and human health.

## AFTER EPUB

[This article was published on 29 November 2024 with errors in Materials and Methods and Fig. 6. The errors were corrected in the current version, posted on 07 April 2025.]

## SUPPLEMENTAL MATERIAL

Supplemental figures spectrum.01983-24-s0001.docx Fig. S1 , S2 and S3.

Table S1 spectrum.01983-24-s0002.xlsx Primers used in this study.

ASM does not own the copyrights to Supplemental Material that may be linked to, or accessed through, an article. The authors have granted ASM a non-exclusive, world-wide license to publish the Supplemental Material files. Please contact the corresponding author directly for reuse.
