# Supplementary material for: Multiomics comparative analysis of feces AMRGs of Duroc pigs and Tibetan and the effect of fecal microbiota transplantation on AMRGs upon antibiotic exposure
Source: Microbiol Spectr. 2024 Nov 29;13(5):e01983-24. doi: 10.1128/spectrum.01983-24 (PMC12054024; doi:10.1128/spectrum.01983-24)
Supplement: Supplemental figures — Fig. S1 , S2 and S3. [file spectrum.01983-24-s0001.docx]

Multi-omics comparative analysis of feces ARGs of Duroc pigs and Tibetan and the effect of fecal microbiota transplantation on ARGs upon antibiotic exposure

Tao Wang^1,2^, Yuheng Luo^1,2^, Xiangfeng Kong^3^, Bing Yu^1,2^, Ping Zheng^1,2^, Zhiqing Huang^1,2^, Xiangbing Mao^1,2^, Jie Yu^1,2^, Junqiu Luo^1,2^, Hui Yan^1,2^, Jun He^1,2*^

^1^Animal Nutrition Institute, Sichuan Agricultural University, Chengdu, Sichuan 611130, P. R. China

^2^Key Laboratory of Animal Disease-resistant Nutrition, Chengdu, Sichuan 611130, P. R. China

^3^Institute of Subtropical Agriculture, Chinese Academy of Sciences, Changsha, Hunan 611130, P. R. China 410125, P. R. China

*Corresponding author: Institute of Animal Nutrition, Sichuan Agricultural University, Chengdu, Sichuan 611130, P. R. China; Tel: +86-13419354223, Fax: +86-28-86291781, Email: [hejun8067@163.com](mailto:hejun8067@163.com).


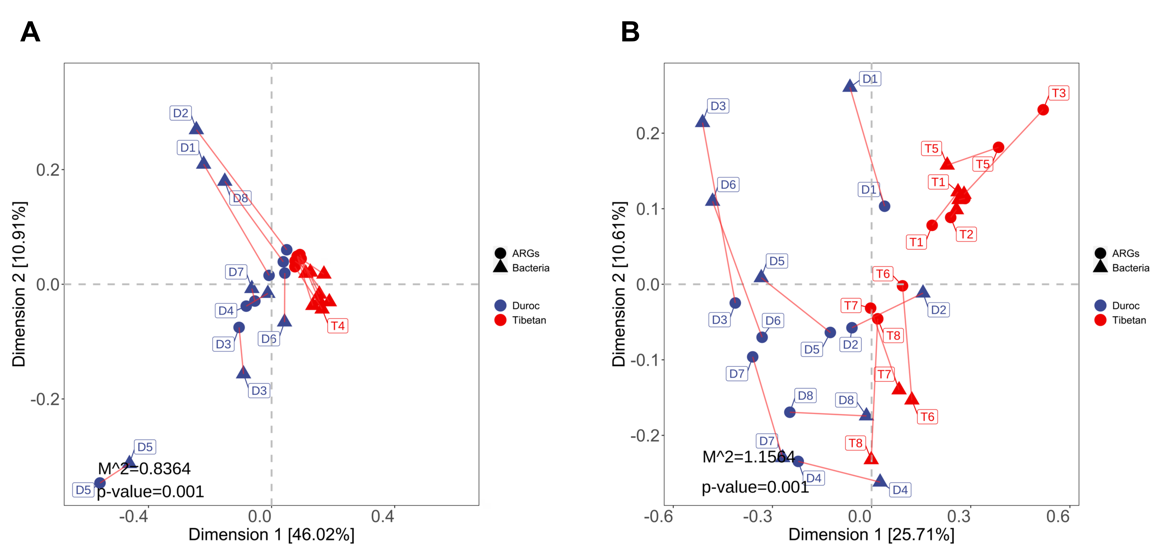


Supplement Figure 1: Abundance associations between gut bacterial species and ARGs were found by analyzing metagenomes (A) and metatranscriptomes (B) through Procrustes.


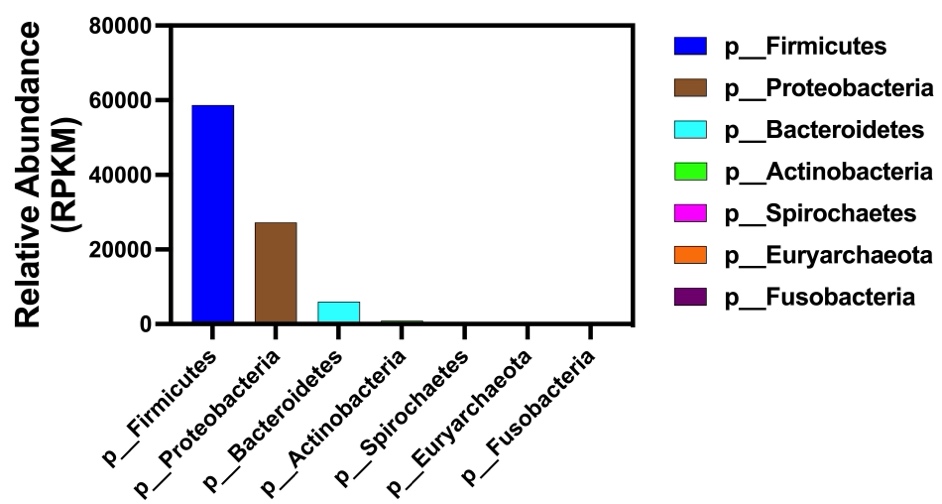


Supplement Figure 2: Relative abundance of microorganisms at different phylum levels.


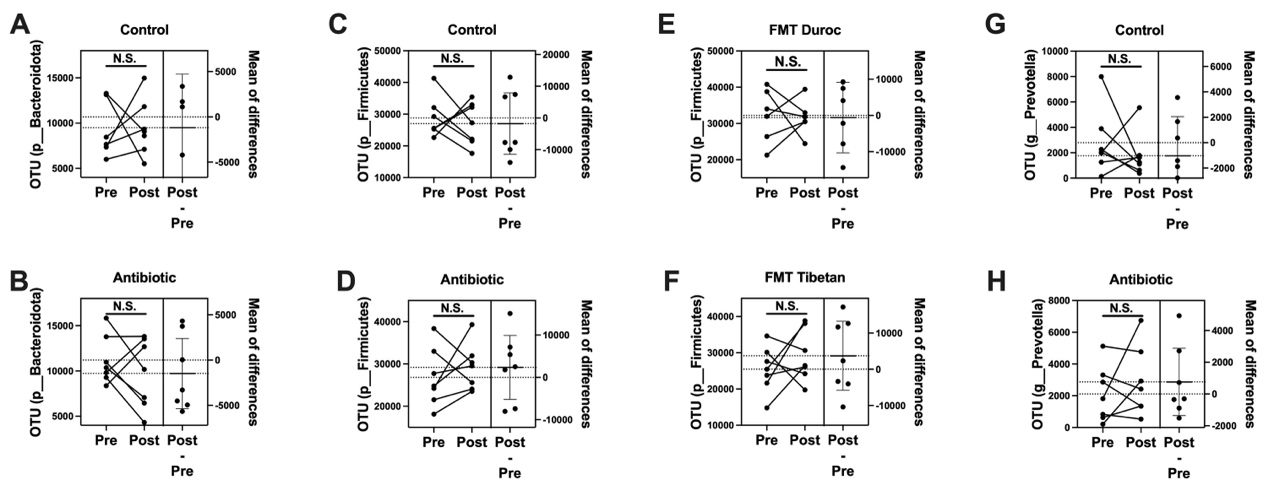


Supplement Figure 3: OUT levels of Bacteroidota (A, B), Firmicutes (C-F) and Prevotella (G, H) in different groups before and after fecal flora transplantation.
